# Impact of angiotensin system inhibitors on esophageal cancer survival^☆^

**DOI:** 10.1016/j.sopen.2020.08.001

**Published:** 2020-09-16

**Authors:** Xuanji Wang, Patrick Sweigert, Emanuel Eguia, M. Alyssa Varsnik, Christen R. Renz, Weston A. Terrasse, Madeline Gauthier, Gerard Aranha, Lawrence M. Knab, Gerard Abood

**Affiliations:** Department of Surgery, Loyola University Medical Center, Maywood, IL

## Abstract

**Introduction:**

Angiotensin system inhibitors are associated with improved prognosis in patients with gastrointestinal and hepatobiliary cancers. Data suggest that renin–angiotensin system signaling stimulates the tumor's immune microenvironment to impact overall survival. The goal of this study is to investigate the role of angiotensin system inhibitor use on the overall survival and disease-free survival of esophageal cancer patients.

**Methods:**

Retrospective review of esophagectomy patients with esophageal adenocarcinoma and squamous cell cancer at a single institution tertiary care center from 2007 to 2018 was performed. Outcomes include overall survival and disease-free survival. Patient characteristics were compared with *t* test and *χ^2^* test. Survival was analyzed with Kaplan–Meier and Cox proportional-hazards regression.

**Results:**

One hundred seventy-one patients were identified and 123 underwent esophagectomy for cancer. No significant differences in patient demographics were found between angiotensin system inhibitor users and non–angiotensin system inhibitor users except for the rates of hypertension (40% vs 94%, *P* < .01) and diabetes (16% vs 47%, *P* < .01). Distributions of tumor neoadjuvant therapy, adjuvant therapy, pathology, staging, margins, and surgical approach were similar. Postoperatively, there was no difference in major adverse cardiovascular events or infection rates. This study did not find any differences in overall survival and disease-free survival between angiotensin system inhibitor users and non–angiotensin system inhibitor users.

**Conclusion:**

Angiotensin system inhibitors have been shown to improve survival and decrease relative risk for several types of cancers; however, our data do not support the same effect on esophageal cancer patients undergoing curative intent surgery. Further research is needed to investigate potential nuances in angiotensin system inhibitor dose, chronicity of use, esophageal pathology, and applicability to nonsurgical candidates.

## INTRODUCTION

Esophageal cancer is the eighth most common malignancy worldwide and represents a highly morbid disease with poor survival [1]. Existing treatment recommendations for localized esophageal and esophagogastric junction cancers include use of preoperative chemoradiation or perioperative chemotherapy [[2], [3], [4]]. Despite multimodal therapy, the 5-year survival is 45% for local disease, 20% with locally advanced disease, and 5% in metastatic disease [5]. Poor long-term outcomes emphasize the demand for maximizing medical and surgical treatment in these patients.

The renin–angiotensin aldosterone system and its physiologic effect on blood pressure, vasoconstriction, fluid homeostasis, and electrolyte recycling have been well described. However, recent investigators have identified mechanisms by which the renin–angiotensin aldosterone system impacts the tumor cell microenvironment [6]. Activation of the renin–angiotensin aldosterone pathway has been shown to augment pathways leading to an optimal tumor growth environment via proinflammatory, proangiogenic, and apoptotic signaling pathways involving angiotensin II, transforming growth factor-*β*, epidermal growth factor, vascular endothelial growth factor, and tyrosine kinase [6,7]. Investigators have identified these pathways as potential novel therapeutic targets [[8], [9], [10]]. Observational studies have demonstrated an association between the use of angiotensin system inhibitors (ASIs) and overall survival (OS) in multiple cancer types including genitourinary, gastrointestinal, and hepatobiliary cancers [11]. Specifically, ASI use has been proposed as an adjunct to standard adjuvant treatment algorithms in patients with advanced pancreatic cancer due to an association with improved OS and disease-free survival (DFS) [12].

The specific mechanism of ASIs in esophageal cancer is poorly understood. In vitro and in vivo studies have respectively shown that ASI inhibits the proliferation of esophageal cancer cells via blockade of the G0 to G1 cell transition, whereas intraperitoneal injection of an ASI in mice leads to a significant reduction in tumor growth and altered miRNA expression [13]. Few studies have connected the implications of the animal model studies with clinical outcomes of ASI use in esophageal cancer survival.

We conducted a retrospective analysis of patients with histologically confirmed esophageal adenocarcinoma and squamous cell cancer (SCC) who underwent curative-intent esophagectomy at a single tertiary medical center from 2007 to 2018. The aim of our study was to determine if there is an association between ASI use and long-term outcomes including OS and DFS. Given the effects of ASIs in other gastrointestinal malignancies, we hypothesized that ASI use may be associated with increased survival in esophageal cancer patients.

## METHODS

### Study Design and Patient Population

The study was approved by the Loyola University Chicago review board (LU# 211937). Adult patients with histologically confirmed invasive esophageal carcinoma (adenocarcinoma or SCC) who underwent esophagectomy at a single tertiary care center from 2007 to 2018 were retrospectively evaluated. Patients with head and neck SCC, Barrett esophagus, and metastatic disease were excluded from the study. Patient, tumor, and treatment characteristics were collected. Patients taking either angiotensin-converting enzyme inhibitor (ACEi) or angiotensin II receptor blocker (ARB) at the time of surgery were classified as ASI users. Time of diagnosis was considered the date of surgery or date of tissue collection. Surgical approach included minimally invasive techniques (laparoscopic and robotic) versus open. Clinical stage and pathological stage were categorized using American Joint Committee on Cancer Eighth Edition [14]. Adverse postoperative events were categorized as major adverse cardiovascular events (stroke, myocardial infarction, pulmonary embolism, and cardiac arrest) and infections (pneumonia, sepsis, septic shock, and *Clostridium difficile* colitis). Perioperative mortality was defined as death within 90 days of surgery.

OS was calculated from the time of diagnosis to the time of death or time of last contact. DFS was calculated from the time of diagnosis to the time of recurrence, death, or last contact.

### Statistical Analysis

Patient characteristics were compared with *t* test for continuous variables or *χ^2^* test for categorical variables. Survival was analyzed with Kaplan–Meier and Cox proportional-hazards regression. Two-sided tests of significance were used. Statistical analyses were performed using R on RStudio Team (2015).

## RESULTS

**Fig 1:**
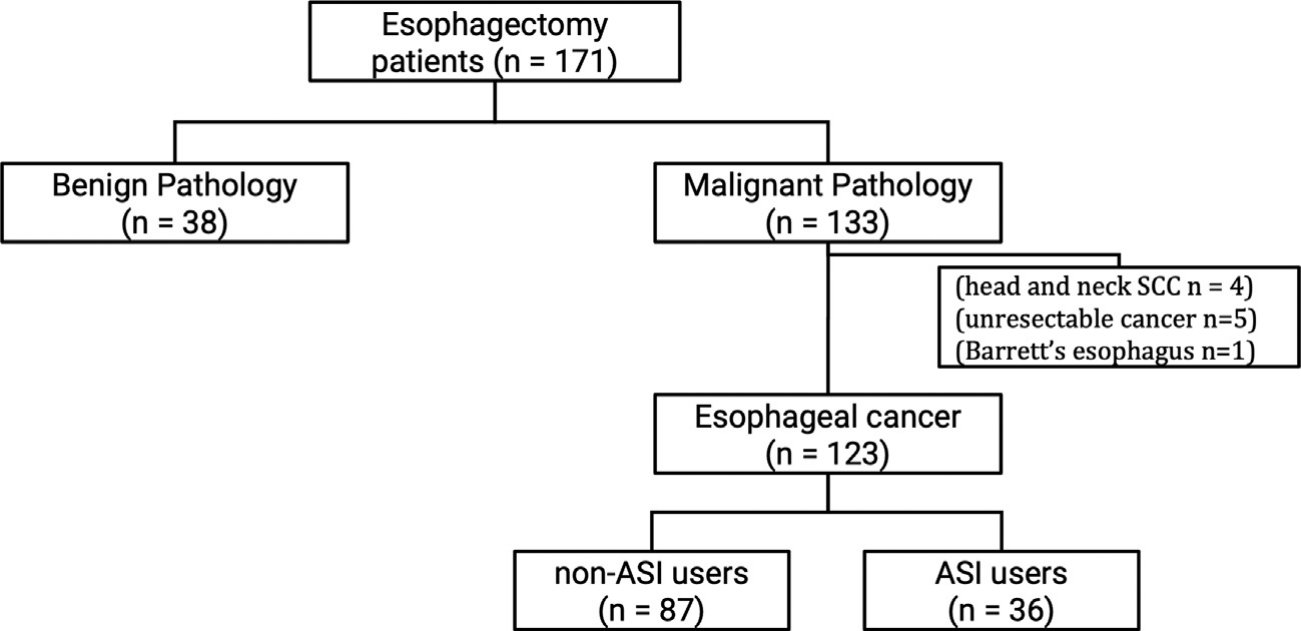
Inclusion and exclusion criteria. One hundred seventy-one patients underwent esophagectomy. Thirty-eight were excluded for benign pathologies. Ten were excluded for head and neck SCC, unresectable cancer at the time of surgery, or Barrett esophagus. Of the 123 patients with esophageal cancer who underwent esophagectomy, 87 were non-ASI users and 36 were ASI users.

A total of 171 patients underwent esophagectomy over the study period, although ultimately 123 were included in the study. Forty-eight patients were excluded (see Fig 1). There were 36 ASI users (29%) and 87 non-ASI users (71%) who were followed for a mean of 27.1 months.

In comparison to non-ASI users, ASI users had higher rates of hypertension (40% vs 94%, *P* < .01) and diabetes (16% vs 47%, *P* < .01). There were no significant differences between patient factors including age, sex, and smoking status. Patient comorbidities such as chronic obstructive pulmonary disease, cardiac disease, and other malignancies were at similar rates. Clinical factors such as tumor location, clinical stage, pathological stage, positive margins, and neoadjuvant and adjuvant therapy were also similar between the 2 groups. A summary of the results can be found in Table 1. Finally, postoperative risk for major adverse cardiovascular events and infections were also not significantly different between non-ASI users and ASI users (Table 2).

**Table 1:** Patient characteristics

	*No ASI (*n *= 87)*	*ASI (*n *= 36)*	P *value*
Patient factors			
Age (average)	64	66	.39
Sex, male, % (*n*)	78% (68)	89% (32)	.26
Current smoker	16% (14)	25% (9)	.37
Disease factors			
Hypertension	40% (35)	94% (34)	<.01
Diabetes	16% (14)	47% (17)	<.01
COPD	3% (3)	3% (1)	1
Cardiac disease (CAD, arrhythmia, valvular)	21% (18)	22% (8)	1
Other malignancies	18% (16)	17% (6)	1
Tumor location	-	-	.86
Cervical/upper	3% (3)	6% (2)	
Mid	9% (8)	8% (3)	
Distal	87% (76)	86% (31)	
Clinical stage			.12
I	11% (10)	14% (5)	1
II	8% (7)	19% (7)	.18
III	55% (48)	61% (22)	1
IV	18% (16)	6% (2)	.09
Pathological stage			.38
I	35% (31)	47% (17)	.32
II	25% (22)	22% (8)	.90
III	33% (29)	31% (11)	.93
IV	6% (5)	0	.33
Pathology, adenocarcinoma, % (*n*)			1
Adenocarcinoma	91% (79)	89% (32)	
Squamous cell carcinoma	9% (8)	11% (4)	
Positive margins	10% (9)	0	.10
Treatment factors			
Surgical approach, laparoscopic, % (*n*)	43% (37)	28% (10)	.15
Neoadjuvant therapy	74% (64)	75% (27)	1
Adjuvant chemotherapy	16% (14)	19% (7)	.85
Adjuvant radiation	8% (7)	8% (3)	1

CAD, coronary artery disease; COPD, chronic obstructive pulmonary disease.

**Table 2:** Secondary outcomes

	*No ASI (*n *= 87)*	*ASI (*n *= 36)*	P *value*
Major adverse cardiovascular events	15% (13)	14% (5)	1
Infections	9% (8)	22% (8)	.10

**Fig 2:**
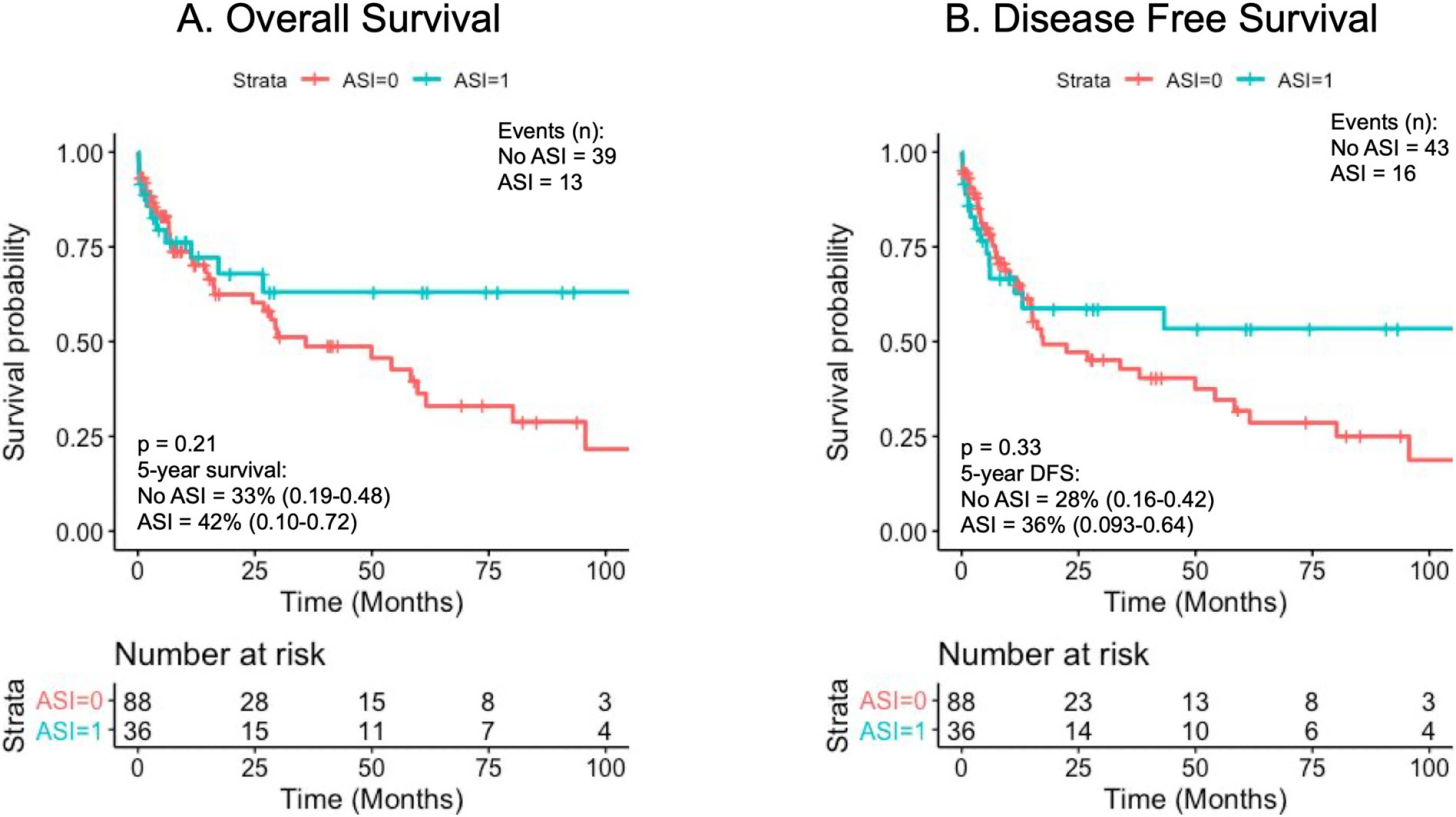
Kaplan–Meier analysis and Cox proportional-hazards regression on survival for non-ASI users (red) versus AS (blue). A, Overall survival: HR = 0.66, CI 0.35-1.25. B, Disease-free survival: HR = 0.75, CI 0.42–1.34, *P* = .33.

The median OS for ASI users compared to non-ASI users was 122.7 vs 35.9 months; however, this did not reach statistical significance (hazard ratio [HR] = 0.66, confidence interval [CI] 0.35–1.25, *P* = .21). Similarly, in the DFS analysis, ASI users had a median DFS of 122.7 months compared to 17.4 months for the non-ASI users (HR = 0.75, CI 0.42–1.34, *P* = .33). Kaplan–Meier survival curves for OS and DFS can be found in Fig 2.

**Fig 3:**
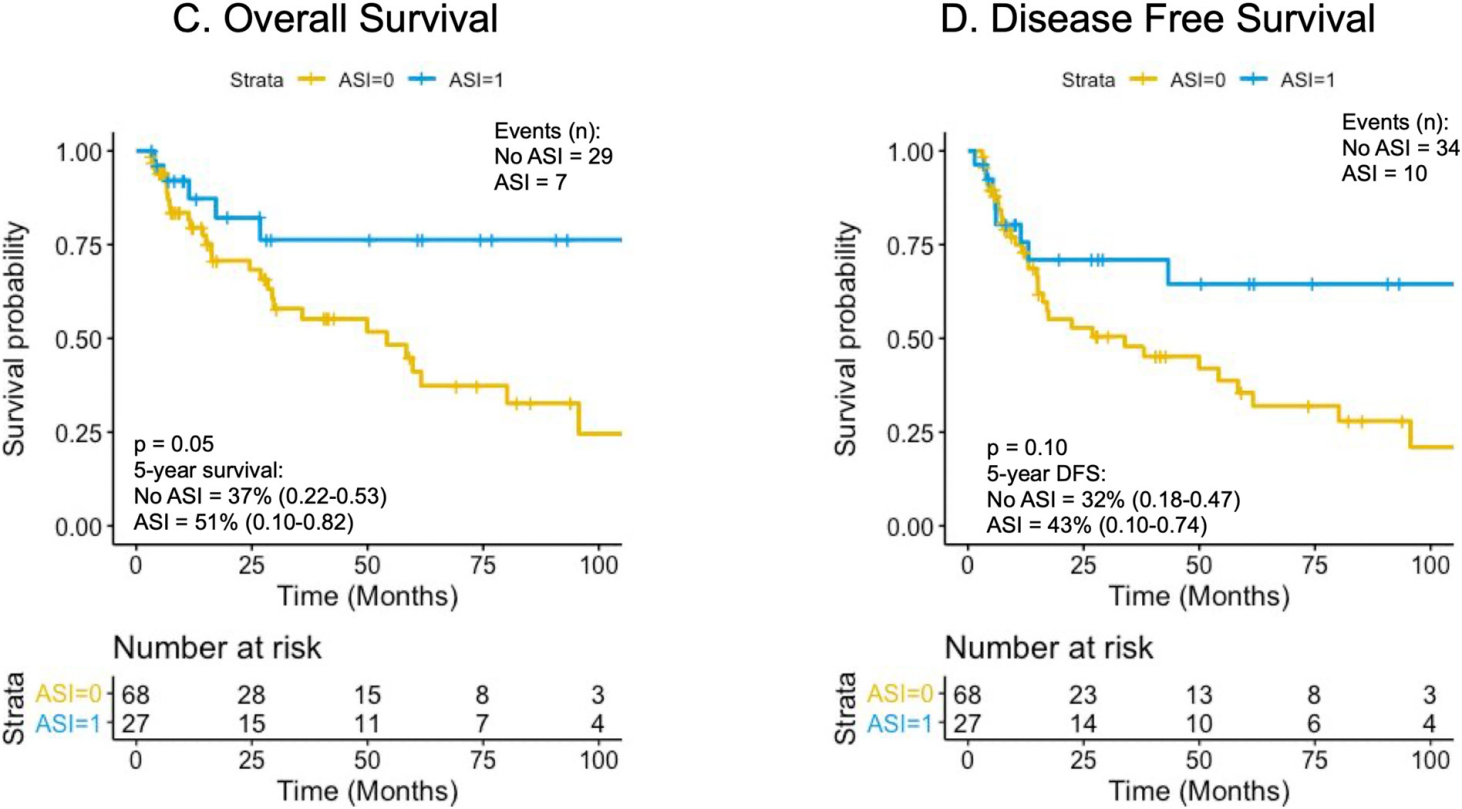
Kaplan–Meier analysis and Cox proportional-hazards regression survival for non-ASI users (yellow) versus ASI users (blue) adjusted to exclude 90-day perioperative mortality. A, Overall survival: HR = 0.44, CI 0.19–1.0, *P* = .05. B, Disease-free survival: HR = 0.55, CI 0.27–1.12, *P* = .10.

A survival analysis was also conducted excluding 90-day mortality. There were 27 ASI users (29%) and 67 non-ASI users (71%). The results showed that median OS was 124.4 vs 54.2 months (HR = 0.44, CI 0.19–1.0, *P* = .05), whereas the median DFS was 122.7 vs 33.9 months (HR = 0.55, CI 0.27–1.12, *P* = .10). Kaplan–Meier survival curves for 90-day mortality-adjusted OS and DFS can be found in Fig 3.

## DISCUSSION

This study retrospectively analyzed the effect of ASI use on survival in patients with esophageal adenocarcinoma and SCC who underwent curative intent esophagectomy. There is limited research evaluating the effects of ASI use in esophageal cancer, particularly in those with adenocarcinoma, which is the most common pathology in the Western population. This study did not demonstrate a significant difference in OS or DFS between the ASI and non-ASI groups. The exclusion of patients with perioperative mortality did not alter these results.

Few studies have examined the role of ASI as an adjunct to existing therapy for esophageal cancer. In patients with esophageal adenocarcinoma, ASI administered in parallel with chemoradiotherapy was found to be superior to chemoradiotherapy alone in terms of OS and DFS [15]. The data suggest that ASI may improve drug and oxygen delivery to tumors, thus enhancing chemotherapeutic effects [16]. Our data show a trend in this direction, although it was not statistically significant. Similar results have been shown in non–small cell lung cancer and pancreatic cancer [17,18]. These clinical associations lack molecular-level evidence showing how ASIs affect the therapeutic pathways of the chemotherapy agents.

This study has several limitations. Primarily, our investigation of ASI use on esophageal cancer is limited to a single-institution cohort. We had a limited number of patients and therefore could be underpowered to determine a difference in survival. Furthermore, the patient selection is skewed toward those who were deemed medically fit enough to undergo esophagectomy. Patients with significant medical comorbidities, unresectable disease, or metastatic disease may still benefit from ASI use to prolong survival, but they were not analyzed in this study.

Another limitation was use of the electronic medical record and difficulty obtaining details about the duration of medication use. Sjoberg et al highlight that there may be some differences in benefit of ASI to reduce risk of esophageal cancer based on duration of therapy (less than 3 years vs greater than 3 years) as well as dose-dependent benefits of ASI [19]. A British cancer registry study suggests that the most significant reduction in cancer-specific mortality among ARB users is in fact with at least 2 years of use [20]. Based on discharge summaries, 34% of our patients were taken off of their ASI during their hospital stay, however it was difficult to determine how many resumed ASI in the postoperative period. Although we expected most patients to resume their normal preoperative medications eventually, this could be a confounding factor. Analysis of the data based on patients taking ASI upon discharge was not meaningful given the small number of patients.

Finally, survival was defined using all-cause mortality instead of cancer-specific mortality because not all causes of death were captured by the electronic medical record. Given the aggressive nature of esophageal cancer, all-cause mortality was used to extrapolate overall survival. A prospective, multicenter study would better capture these nuances to address the aforementioned limitations.

## Conclusion

In conclusion, this study investigated the effects of ASI use in esophageal cancer patients at a single institution. We found no overall or disease-free survival advantage associated with ASI use in esophagectomy patients. Angiotensin-converting enzyme inhibitor and ARB have been extensively studied with hopes of finding an inexpensive and pharmacologically safe therapy to supplement existing cancer treatment algorithms. Further studies examining the effect of ASI on esophageal cancer as well as chemotherapeutic pathways are warranted to better understand the pharmacologic impact of ASIs and to determine if they have an impact on cancer-specific survival.
